# Measuring optimal psychological states: Proposal of two brief versions to measure flow and clutch in athletes

**DOI:** 10.3389/fpsyg.2023.1066494

**Published:** 2023-01-20

**Authors:** Alejandro Sánchez Vara, José L. Chamorro, Ruben Moreno Castellanos, Saul Alcaraz

**Affiliations:** ^1^School for Doctoral Studies and Research, Universidad Europea de Madrid, Madrid, Spain; ^2^Faculty of Sports Sciences, Universidad Europea de Madrid, Madrid, Spain; ^3^Hum-878 Research Team, Department of Psychology, Health Research Centre, University of Almería, Almería, Spain; ^4^Sport Research Institute UAB, Autonomous University of Barcelona, Barcelona, Spain

**Keywords:** flow, clutch, estados óptimos psicológicos, versión corta, validación de cuestionario flow, optimal psychological states, short versions, scale validation

## Abstract

**Background:**

The study of sports experiences with high levels of performance has led to the observation of two psychological states categorized as optimal, denominated flow and clutch. The objective of this study is to design and validate two brief scales version to measure flow and clutch.

**Methods:**

Following the MIMO (Maximum Information, Minimum Discomfort) protocol, three studies are carried out: In Study 1, we developed the short versions of the questionnaires based on theory-driven and data-driven criteria; in Study 2, we used quantitative criteria to validate the short versions; and in Study 3, we checked the final version of each scale to observe their statistical validity and their relation to other variables.

**Results:**

The new short flow scale is made up of seven items, while the clutch scale is made up of five items. Throughout the three studies, evidence is provided of the internal consistency, invariance of the measurement models and content validity, validity related to the responding process and validity in relation to other variables.

**Conclusion:**

This study provides two short versions to evaluate flow and clutch, which will help to continue advancing in the study of optimal psychological states in sport.

## Introduction

Flow is considered a complex psychological state crucial for the life development of human beings (Csikszentmihalyi, 1992). Although the early research about this phenomenon was conducted in people’s everyday life (Csikszentmihalyi, 1975a,b, 1981), it soon started to be investigated in the sport context and was defined as the optimal state for the sports practice (Chalip et al., 1984; Jackson, 1992; Jackson and Roberts, 1992; Kimiecik and Stein, 1992). Because of the relevance of this state and looking for a deeper understanding of it, Swann et al. (2016a) ran an observation on professional golfers during tournaments. In this research, they found in athletes’ explanations two different psychological states during their best performances. The authors described these two states with the expressions “letting it happen” and “making it happen” (Swann et al., 2016b). They described “letting it happen” as an experience characterized by open goals, less contextual structure and related to exploratory and self-referenced behaviors. They considered this state the equivalent of flow. The other state, “making it happen”, introduces new characteristics from the nature of the objectives and the context, with fixed goals, a more defined structure and the recognition of non-subjective goals related to the context. The researchers considered this second state to be something similar to those performances called “clutch performance”, described by Hibbs (2010), where the athlete can achieve positive results using their own abilities in contexts with high pressure.

Thus, currently, we have two optimal psychological states whose definition contains particular characteristics. On one hand, Flow is the experience of a subjective state that merges within the sport context that challenge the abilities of an individual. The achievement of goals in this state is produced from an exploratory attitude of the individual’s possibilities of action, connecting with their context and flowing with the consecutive application of skills. The original definition of flow describes it as a multidimensional state with nine dimensions: (a) perceived challenges that stretch existing skills, (b) action-awareness union, (c) clear goals, (d) positive feedback, (e) concentration in the task at hand, (f) sense of control in what one is doing, (g) loss of self-consciousness, (h) distortion of time perception, and (i) autotelic experience (Csikszentmihalyi, 1975a,^1990^; Jackson and Marsh, 1996). On the other hand, Clutch is the experience of a subjective state derived from the perception of an increase in contextual pressure on the individual due to time or results demands derived from sports practice. The individual will be able to recognize and manage the stress originated, so that he will raise his physical and mental energy levels to achieve the proposed objectives. Clutch has been defined as a psychological state characterized by (a) complete and deliberate attention, (b) intense physical effort, (c) high ego attention, (d) strong physiological activation, (e) absence of negative thoughts, and (f) automaticity in the use of one’s own abilities (Swann et al., 2016a,2017a,b,^2019^).

The study of the optimal psychological states is relevant for the relation of their characteristics and the best experiences in human beings. While clutch is still under study, flow state has been understood as the intersection between the greatest experiences and peak performance (Jackson and Roberts, 1992; Kimiecik and Stein, 1992; Jackson and Marsh, 1996; Engeser and Rheinberg, 2008; García Calvo et al., 2008; Bakker et al., 2011; Swann et al., 2012; Verner-Filion and Vallerand, 2018; Tse et al., 2019). In any case, flow experience has been considered rare and elusive (Csikszentmihalyi, 1975b,^1986^; Kimiecik and Stein, 1992; Swann et al., 2017a). The researchers have not been able to explain, for example, the different variations in the intensity perception during the experience of this state (Csikszentmihalyi, 1990, 1999; de Nakamura and Csikszentmihalyi, 2014), justifying it based on different factors, depending on the authors or the methodologies (Rheinberg et al., 2003; Swann et al., 2012; Fernández et al., 2015; Stander et al., 2015; Chang, 2016; Sinnett et al., 2020). Although the first conceptualization of flow has been generally accepted and the use of the scales respecting the original nine dimensions generalized, some theoretical and methodological problems have been observed, and some authors have found *anomalies* in the flow research (Rheinberg et al., 2003; Swann et al., 2018). Some examples of these anomalies are the objections to the dimensions of loss of self-consciousness and time distortion dimensions (Jackson and Marsh, 1996; García Calvo et al., 2008), or specific problems in the time distortion dimension in other authors (Jackson and Marsh, 1996; Marsh and Jackson, 1999; Vlachopoulos et al., 2000). Swann et al. (2012) showed it in their systematic review, where only 28.95% of the total athletes in flow research speak about that specific dimension. These authors explained that variations in the context can modify the subjective perception of time (for example, the type of sport). For all that, as we said before, the number of variations in the intensity of the subjective experience of the athletes, and the issues on the causality explanation of the state, there is a need for a better explanation of this new state denominated clutch.

For flow research, different tools were developed during past years. But in the validation process, some instabilities in the conceptualization of flow were observed (see Table 1). After the first qualitative studies to identify flow in sport, (Jackson, 1992, 1995; Jackson and Roberts, 1992), Jackson and Marsh (1996) developed the Flow State Scale (FSS) with 36 items (four items/dimension). That first scale was focused on specific events, such as training or specific competition games. Some variations were introduced later with the objective of studying the dispositional flow, which means the frequency that an athlete can experience flow (*Dispositional Flow Scale*, DFS; Jackson et al., 1998). After some problems in data reliability and the discovery of some conceptual areas that could be improved (Vlachopoulos et al., 2000), Jackson and Eklund (2002) reviewed the FSS and introduced changes in some items, calling the new tool FSS-2. With the objective of being used and modified in sporting contexts and other areas of psychology in different cultures, the FSS was translated into Spanish for García Calvo et al. (2008). Some researchers considered it interesting to work in short forms of the FSS, too. So, Rheinberg et al. (2003), proposed a 10-item short-form (*Flow-Kurzskala* – FKS). Despite some reliability problems in their statistical analysis the authors asserted that all components of the flow experience can be measured with 10 items. In their study, they found two factors (FI & FII) within the scale. They showed that high values in their scale of total flow indicate that both factors (FI & FII) were presented, while, with medium values, accentuations of one factor occurred to the detriment of the other. This means that the perception of one factor did not permit the experience of the other factor at the same time. Jackson et al. (2008) developed a short version with one item for dimension, respecting the original structure definition of the concept and promoting the use of short forms. They defended that the short versions proved to be just as reliable as the long versions, maintaining the validity of the construct and defending its multidimensional explanation with 1 item for each dimension. Despite some issues in their results, they conclude that “psychometric support” is achieved together with “internal consistency and external validity” (p. 29) in relation to the key elements of the construct (Jackson et al., 2008). The authors recommended continuing investigating new short forms.

**TABLE 1:** Flow questionnaires, characteristics, and limitations, over the years.

Name	Reference	Items	Language	Characteristics	Limitations
*Flow State Scale – FSS*	Jackson and Marsh, 1996	36	English	9 factor model, with 4 items for each dimension. Likert scale of 5 points, from 1, strongly disagree to 5, strongly agree.	Lower factor loadings in Transformation of Time and Loss of Self-Consciousness. No clarification if one global factor is valid or must be used always using 9 factors individually. Retrospective approach to data collection. Heterogeneous sample may not generalize to specific subgroups.
*Dispositional Flow Scale - DSS*	Jackson et al., 1998	36	English	Based on FSS, respect 9 factors and 36 items (4 each dimension). Changes in the wording and the tense of phrases designed to assess frequency.	Sample was limited to older athletes, not having control over when participants completed the state assessment. No answer to the global factor to measure flow. Lack of relationship with the motivation scales and variables. Lack of relationship between challenges ratings and flow subscales.
*Flow State Scale-2 – FSS-2*	Jackson and Eklund, 2002	36	English	Original 36-item FFS along with 13 additional items devised as potential replacements. 5-point Likert scale ranging from 1, never to 5, always.	The higher-order factor loadings of time transformation remain relatively weak, being unclear whether the item is problematic. Differences in factor loadings expresses differences in flow experiences, but there is no specific answer on why this can be supported (maybe differences in sports or physical activity). Special problems in the perception of the self. Limited control over when the questionnaires are completed.
*Flow State Scale (Spanish Version)*	García Calvo et al., 2008	36	Spanish	Translation of the original FSS to Spanish. 36 items and 9 factors. 10-point Likert-type scale.	Lowest value in sense of time and loss of self-consciousness, where the authors propose to revise them in future research. Different correlations to motivational variables compare to DFS. Heterogeneous sample produce unexpected relations, with heterogeneous values in its results.
*Flow-Kurzskala* – FKS	Rheinberg et al., 2003	10	German	10 items adapted from the original FSS where selected. 7-point Likert-type scale.	Include new items in the scale that were not related to flow dimensions or construct, that have weak psychometric stability. Not achieve desired fit limits and had cross-loadings and unsatisfactory factors loadings.
*Short Flow Scale - SFS*	Jackson et al., 2008	9	English	9 items, with one item from the four-item measures of each on the nine flow dimensions.	Did not reach acceptable criterion levels of fit across all indices. The situation-specific state short measure requires more discrimination between factors (fit is reduced). Limitations of the self-report and retrospective recall. Problems in validity and reliability and additional research is needed to examine its psychometric properties.

In recent years, the methods used to study clutch have been qualitative methodologies, experimental approaches measuring subjective experiences, introduction of modifications in athletes’ skills or analysis of the competitive results during clutch performances. All these methodologies brought us to the situation of defining clutch as a result of a performance, as an ability, as a specific situation or as a psychological state (Schweickle et al., 2020). In that variety of definitions, clutch has been measured as a skill and/or a performance episode, looking to prove its occurrence and evidence, and the obtained results have been very unsatisfactory. Until today, definitions of clutch still lack specificity and clarity, creating misunderstandings about what this concept is (Schweickle et al., 2020). For all this, the authors of the clutch definition that we follow invite the researchers to develop new questionnaires that can distinguish flow and clutch states, since the methodologies and tools used until today may not be differentiating characteristics from both experiences, being all aspects assigned to flow. This is happening due to the little validity for the discrimination of states that have the current tools (Swann et al., 2017b,^2019^).

The objective of this study is to design and validate two independent short instruments to measure the psychological states of flow and clutch in Spanish. One short form for flow and a new scale, based on the Integrated Model of Flow and Clutch States, for clutch. Our main focus during the development of these scales was to develop instruments that assessed the core aspects of each psychological state. In this line, we wanted to create efficient single-factor measures that were able to capture the most important characteristics of each state.

In addition, short questionnaires improve data quality, as long tools have been found to lead to boredom and apathy (Coste et al., 1997; ; Jackson et al., 2008; Alcaraz et al., 2013, 2020; García-Alba et al., 2021). In addition, it will be following the recommendations of authors who defend that shortening existing tools is better than creating new ones since it saves phases in their creation and the instrument will be familiar for the participants (Coste et al., 1997; Alcaraz et al., 2013, 2020). For that, the Spanish translation of the FSS by García Calvo et al. (2008) will be used to develop a short form for flow. And for clutch, a new scale is going to be developed since there is still no questionnaire to measure this phenomenon as a psychological state in Spanish.

Our process of development and validation of the short forms relies on the Maximum Information, Minimum Discomfort method (MIMO, Alcaraz et al., 2020). This protocol is initially based on Boateng et al., 2018 recommendation to develop new questionnaires and is divided into four phases. In this study, we present three different studies that cover all the stages of MIMO development. Specifically, in Study 1 we selected the initial pool of items based on theory-driven, data-driven and comprehension-related criteria (Phase I), refined the short-form with the evaluation of a panel of experts (Phase II), and assessed the target population’s understanding of the items (Phase III). Subsequently, in Studies 2 and 3 we targeted Phase IV. On the one hand, in Study 2 we assessed the dimensionality and reliability of the questionnaires. On the other hand, in Study 3 we confirmed the factor structure of both questionnaires and provided validity evidence based on relation to other variables. Ethical approval for this investigation was obtained from the University Ethics Committee (reference: CIPI/19/011).

## Study 1: Pilot study. Short-form development

For scale validation, the MIMO strategy (Maximum Information, Minimum Discomfort, Alcaraz et al., 2020) has been followed and Study 1 will complete Phase 1: Initial Item Selection, Phase 2: Expert Group, and Phase 3: Comprehension of Focus Group.

### Method

#### Participants

We obtained qualitative and quantitative data from participants in Study 1 to develop the short forms. Qualitative data came from an expert committee with extensive experience in applied research and sports psychology counseling, all of whom usually work in different Universities in Spain and are part of different research groups of psychology. The other source was a focus group formed by four athletes, three men and one woman, with ages between 18 and 21 years old, all of them practitioners of team sports (football, basketball, volleyball, and handball), with more than five years of experience in regular training and competition, and University students.

Qualitative data came from a sample of 128 young football players belonging to a club in the Community of Madrid (Sample 1). The sample was totally male and ranged in age from 14 to 23 years (*M* = 16.28, *SD* = 2.60). All the players trained regularly (3 weekly training sessions) for 1 hour and 30 minutes and competed in their respective categories (represented by a total of 7) within the Madrid Royal Football Federation (RFFM) championships on weekends. The accumulated experience of the players varied from 2 to 18 (*M* = 8.48, *SD* = 3.82) training seasons.

#### Instruments

For the flow study, we administered the translation of the original version of the FSS (Jackson and Marsh, 1996). For the clutch study, we developed a new scale.

##### Flow State Scale (FSS)

To ensure that the sample responded to the concept of flow, the Spanish version of the FSS translated by García Calvo et al. (2008) was presented. The original version of the FSS was developed by Jackson and Marsh (1996) and is composed of 36 total items, 4 for each of the 9 dimensions, which are answered on a 10-point Likert-type scale, where 1 means totally disagree and 10 totally agree. This translation of the Spanish FSS obtained optimal reliability values, where Cronbach’s alpha reached values above 0.70, and in several cases, above 0.80. In addition, for its translation, a sample had been used in which there were many participants dedicated to the practice of team sports, especially football.

##### Clutch State Scale (CSS)

A scale with six items was developed, one for each characteristic that distinguishes the state of clutch with that of flow, based on the proposal in the work of Swann et al. (2016a). These six items created based on the theoretical development of the clutch characteristics were presented to the focus group for discussion and verification of their understanding. At the same time, the 10-point likert-type scale is maintained, where 1 is totally disagree and 10 totally agree.

#### Procedure

The development of short forms following MIMO criteria explains that in Phase I, Initial Item Selection, the process must respect three conditions for the item selection: theoretical, psychometrics, and comprehension.

Our theory-driven criteria came from a literature review and the advice of an expert committee. After analyzing the original version of the FSS, they were entrusted with a double task: order numerically, under their criteria, which items best represented the definition of each dimension for flow, and, at the same time, provide qualitative justification of the selected order. At this stage, three new item proposals were included for those dimensions in which it was considered that the existing items did not make “reference to the core of the construct” (p. 55, Alcaraz et al., 2020). From these three items, after semantic analysis and discussion, finally were included two related to the dimensions of clear goals (item 3: *It was very clear what I needed to do, and I wanted to achieve*) and sense of control (item 6: *I was feeling a sense of control over my body and mind*). The shortening process continued with the focus group, which was conducted to explore the opinion of our target population. During these interviews, three items selected of the FSS were specially discussed: those related to the dimensions of clear goals, loss of self-consciousness, and distortion of time. In the dimension of clear goals, the first proposal did not distinguish between self-perception and the perception of the coach to define the quality of the performance. In the loss of self-consciousness, there was a misunderstanding between the worried thoughts related to the self and coming from the peers. And in the time distortion, it seems that athletes have two kind of time perceptions that represents different levels. One level, that we can call it *micro*, refers to the temporal perception related to the skills execution. And the second level, more general, that can be called *macro*, refers to the general perception of the time needed to realize the complete the task. These conceptual differences were discussed with the expert committee, and the changes considered relevant and notorious were introduced. The answers were given on a 10-point Likert-type scale.

To measure the clutch experience, following the theoretical principles exposed in Swann et al. (2016a), a new scale was developed. Being a first version of the scale in Spanish for the study of the new theoretical construct developed in English, the characteristics proposed by the original authors were translated into Spanish. The Spanish version of the clutch characteristics was analyzed and compared with the flow dimensions by the expert committee: changes in some lexical and meaning nuances were introduced with the objective to differentiate both states in the same questionnaire. At the same time, several versions of the scale introduction were developed, introducing the specific characteristics that distinguish flow from clutch and that are originated in variations in the sporting context. Thus, a 6-item scale was generated in which each item responded to each of the characteristics proposed by Swann et al. (2016a). The answers were given on a 10-point Likert-type scale, thereby maintaining the structure of the FSS. To help participants identify clutch as a state other than flow, the scale was presented with a different introduction to flow based on its phenomenological characteristics. For clutch, the emphasis was placed on the contextual variation that affects the perception of the experience by the individual, as this state was related to a sudden assessment of situational demands due to a change in them. For this reason, in the scale presentation, the participant is reminded that *“during a training session or match there are variations in the context due to external causes”*, exposing several possibilities below (result, orders from the coach or pressure from the public) and asking if, based on these situational changes, *“you have changed the way you face your participation”.*

The psychometric criteria were obtained from the data of the original questionnaire, together with the data of its application in Sample 1. In this phase, the instruments were administered using paper and a pen at the club’s facilities, where they had a meeting room used for the application of the questionnaire. All minors had previously been given the informed consent document, which was returned with the signature of their parents or guardians before completing the questionnaire. The objectives and characteristics of the study were reported, as well as the confidentiality and anonymity of the responses, remembering that their participation was voluntary. The questionnaires were always kept and guarded by the principal investigator of the study. Once data were collected, data-driven criteria were obtained from results of internal reliability and internal structure. More information about these analyses can be found in results section. The data-driven criteria helped us to determine the items that conform the short-form questionnaire and observe the validity of the clutch scale.

In the clutch scale, no conceptual issues were found, and focus group found it clear and comprehensible.

### Results

As a result of the procedure conducted with the expert committee, the focus groups with the target populations, and the research meetings, we obtained a great number of criteria to select the items that would be included in flow and clutch questionnaires. Such criteria were complemented with the data collected with Sample 1. First, we obtained the descriptive statistics for each item and dimension and then, we assessed the correlation between dimensions. As can be observed in Table 2 for flow and Table 3 for clutch, almost all correlations between dimensions and characteristics are statistically significant. Only in the dimension distortion of time, some relations were smaller and not significant, although they were being positive as expected. Specifically, such smaller relationships are only observed with three dimensions: positive feedback, concentration in the task at hand, and loss of self-consciousness. Related to clutch items, all correlations are positive, as expected, and significant, being ≥ 0.377. The lowest correlation is observed between automaticity of skills and heightened arousal.

**TABLE 2:** Correlations between items for FSS shortening.

	Balance	Union	Clear goals	Feedback	Concentration	Control	Loss of self	Time	Autotelic
**Balance**									
Union	0.197**								
Clear goals	0.401**	0.291**							
Feedback	0.489**	0.298**	0.335**						
Concentration	0.250**	0.256**	0.493**	0.462**					
Control	0.520**	0.415**	0.546**	0.460**	0.371**				
Loss of self	0.298**	0.179**	0.227**	0.361**	0.541**	0.345**			
Time	0.297**	0.217**	0.282**	0.174	0.123	0.272**	0.158		
Autotelic	0.263**	0.314**	0.303**	0.398**	0.361**	0.289**	0.203*	0.263**	

The nine dimensions of the original FSS scale: (a) Challenge-skill balance, (b) Action-awareness merging, (c) Clear goals, (d) Unambiguous feedback, (e) Concentration on the task at hand, (f) Sense of control, (g) Loss of self-consciousness, (h) Transformation of time, (i) Autotelic experience.

*p < 0.05; **p < 0.01.

**TABLE 3:** Item correlations between items for clutch.

	Complete focus	Intense effort	Heightened awareness	Heightened arousal	No negative thoughts	Automaticity of skills
**Complete focus**						
Intense effort	0.643**					
Heightened awareness	0.595**	0.582**				
Heightened arousal	0.523**	0.527**	0.534**			
No negative thoughts	0.676**	0.564**	0.559*	0.491**		
Automaticity of skills	0.394**	0.431**	0.389**	0.377*	0.417**	

*p < 0.05; **p < 0.01.

### Discussion

In Study 1, we developed the short forms by using theory-driven as well as data-driven and comprehension criteria. Initial short forms for flow and clutch were created (Version 0). A future study was needed to validate these new short forms.

## Study 2: Preliminary psychometric validation of the short forms

Following with the MIMO structure (Alcaraz et al., 2020), in Study 2, we developed the first part of Phase IV, Evaluation of the Short Version. With the application of the two scales, its statistical analysis is developed, in which its reliability and validity are assessed together with the relationship between dimensions and characteristics.

### Participants

Sample 2 consisted of 286 young soccer players, aged between 14 and 18 years, (*M* = 16.56, *SD* = 0.94), and of whom 217 (75.9%) were male and 69 (24.1%) females. These players were distributed in a total of 16 different teams within 12 different clubs located in different cities of Spain and covering up to nine different categories, between men’s and women’s football. The experience of the group of players of both sexes ranged between 1 and 13 seasons (*M* = 7.68, *SD* = 3.28) of training.

In addition, the aim was to introduce gender differences in the sample, since differences between boys and girls have been observed in other studies on optimal psychological states (Elbe et al., 2010; Chang, 2016).

#### Instruments

##### Flow state short-scale (FSSS)

The short version obtained from the quantitative and qualitative analysis of Study 1 was used (Version 0). This process led us to develop a short flow scale of nine items, one for each dimension that is part of the state. The scale was answered in the same 10 points Likert scale, as in Study 1, respecting the original construction.

##### Clutch state scale (CSS)

After the analysis of the data in Study 1 (Version 0), this scale included six items, each of them related to a dimension of clutch. The stem of the questionnaire was “In some specific moment of training/match.”. The scale was answered in the same 10 points Likert scale, as in Study 1.

### Procedure

The different sports facilities of the participating clubs were visited, and the questionnaires were administered using paper and a pen at the same venues. The main researcher was always with the different teams solving all doubts that could arise when reading and completing the scales. The questionnaires never took more than 20 minutes to answer, and the technical staff of the teams helped in the organization. All minors had previously been given the informed consent document, which was returned with the signature of their parents or guardians before completing the questionnaire. The objectives and characteristics of the study were reported, as well as the confidentiality and anonymity of the responses, remembering that their participation was voluntary.

### Data analysis

Data were analyzed using SPSS version 17 and Mplus version 7.4 (Muthén and Muthén, 2015). First, preliminary analyses included the detection of missing values and the analysis of data distribution. Subsequently, we tested and refined the measurement models of the Flow State Short-Scale (*FSSS*) and Clutch State Scale (*CSS*). As our focus was to develop efficient questionnaires that assessed the core aspects of each psychological state, both instruments were expected to fit single-factor structures. However, to obtain evidence supporting such assumption, we first conducted exploratory factor analyses (EFA) to assess whether a single-factor structure was more suitable than a multi-factor structure. Varimax was selected as rotation method. Such analyses were conducted using IBM SPSS statistics 23.

Then, the single-factor structure of both instruments was examined with Mplus using a Confirmatory Factor Analysis (CFA) approach. To that end, we tested a series of single-scale CFA models to refine the Version 1 of both questionnaires. We started from an initial 9-item measurement model for the FSSS and from a 6-item measurement model for the CSS, and then we deleted the items that failed to produce sufficient measurement requirements. As the main idea was to assess the core aspects of flow and clutch, all measurement models were expected to fit a single-factor structure. We considered items for deletion when they included target standardized factor loadings of < 0.40. We also inspected modification indices to identify problematic items. Finally, we considered theory-driven criteria when necessary. All the items shared similar scrutiny and were deleted after we reached consensus. This process resulted in the Version 2 of FSSS and CSS.

All CFA models were estimated based on the Maximum Likelihood (ML) estimator. Different fit indices were used to test the fit of the CFA measurement models to the data: χ^2^ statistic, Comparative Fit Index (CFI; Bentler, 1990), Tucker–Lewis Index (TLI; Tucker and Lewis, 1973), and root mean square error of approximation (RMSEA; Steiger and Lind, 1980) including its 90% confidence intervals (CI). A non-significant χ^2^ value indicates a close fit between the observed and the expected model values. The threshold of acceptable fit for the RMSEA is ≤ 0.08 (for an excellent fit, £0.06; Browne and Cudeck, 1993). Additionally, CFI and TLI values > 0.95 are considered as indicators of excellent fit (Hu and Bentler, 1999).

To assess the reliability of the questionnaires, we used McDonald’s (1970) coefficient omega (ω), which is computed from the standardized parameter estimates of the model. Finally, concurrent validity of both questionnaires was analyzed via a deattenuated correlations between their latent factors. To do so, the two latent factors were included in a single measurement model to compute these correlations.

### Results

Percentages of missing data were < 1.4% for all the items. According to Graham’s (2009) criterion, our missing data had no psychometric effects on data analyses. Subsequently, we conducted an EFA with each questionnaire to test whether a single-factor structure was more convenient than a multi-factor structure. In the case of FSSS, the results of the EFA returned two factors with an eigenvalue > 1. However, the rotated solution showed one factor with 7 items and a second factor with only two items (items 2 and 8; further explanation on these two items is provided in the next paragraph). The percentages of explained variance were as follows: Component 1 = 36.8%, Component 2 = 13.2%. Regarding the CSS, the EFA results also supported the preference for a single-factor structure. In fact, only one factor presented an eigenvalue > 1 (% of variance = 49.7%). Further description of these results is available from the first author upon request.

Then, a series of single-factor CFA models were tested to refine the measurement model of both questionnaires. For the FSSS, the *a priori* 9-item model showed a fit to the data of χ^2^(*df*) = 73.247 (27), *p* < 0.001, RMSEA [90% CI] = 0.077 [0.056–0.099], CFI = 0.912, TLI = 0.882 (Table 4). Thus, deletion of some of the items appeared to be necessary. Specifically, the model significantly improved when Items 2 (*Things just seemed to be happening automatically*) and 8 (*At times, it almost seemed like things were happening in slow motion*) were removed from the analysis. Those items were considered for deletion as they presented insufficient factor loadings in the initial model (l_item 2_ = 0.193, l_item 8_ = 0.143). In addition, deletion of those items was also supported by the previous exploratory factor analysis. From a theoretical standpoint, problems with the items that refer to the dimension of Transformation of time were observed from the very beginning of flow research. In the same FSS development (Jackson and Marsh, 1996), the authors considered that dimension “*less universally important*” (p.30), due to less support from the athlete’s experiences and factor loadings. Swann et al. (2012) confirmed that point in their systematic review about flow: Transformation of time was the last dimension in terms of athlete’s citation (only 28.95%, when the first, related to concentration, achieved 80.7%). In the same line, in the Spanish version of the FSS (García Calvo et al., 2008), those two dimensions were the dimensions with the lowest values (Transformation of time and Balance of action and awareness,0.42,0.59, respectively). In other research using Spanish scales with swimmers (López-Torres et al., 2007), the dimension Balance of action and awareness is the 6*^th^*, out of the 9 dimensions, in terms of the experience score when athletes describe flow. It seems that the significance of the sense of union between action and awareness could be different in the Spanish context.

**TABLE 4:** Comparison among CFA measurement models (Flow state short-scale).

Model	χ*^2^*(*df*)	*p*	RMSEA (CI 90%)	CFI	TLI
**Study 2**					
Original 9-item Flow State Short-Scale	73.247 (27)	<0.001	0.077 (0.056 –0.099)	0.912	0.882
Deletion of item 2 and item 8, item 3 (Version 2)	25.409 (14)	0.031	0.053 (0.016 –0.086)	0.976	0.964
**Study 3**					
Version 2	79.207 (14)	<0.001	0.093 (0.074 –0.114)	0.932	0.898

The item-deletion process resulted in a 7-item, single-factor FSSS, which showed a good fit to the data: χ^2^(*df*) = 25.409 (14), *p* = 0.031, RMSEA [90% CI] = 0.053 [0.013–0.086], CFI = 0.976, TLI = 0.964. In addition, all the items included in this model showed satisfactory factor loadings > 0.40 (range = 0.487–0.697; see Supplementary Table 1 in Supplementary material) and no modification indices suggested further item deletion. The data distribution and factor loadings for the retained items are available in Supplementary Table 1 (Supplementary material). Regarding the reliability of this scale, McDonald’s (1970) coefficient omega was excellent^1^: Ω = 0.789. From this point forward, we refer to the 7-item, single-factor FSSS as FSSS –Version 2.

Regarding the CSS, the analysis began with a 6-item single-factor model (see Table 5). This measurement model provided an acceptable fit to the data: χ^2^(*df*) = 30.649 (9), *p* < 0.001, RMSEA [90% CI] = 0.092 [0.057 –0.129], CFI = 0.954, TLI = 0.924. However, Item 6 (*I wasn’t thinking in the execution, I was focus on the general context*) presented a factor loading below 0.40 and were considered for deletion (l = 0.290). Considering the theory-driven criteria, there was a problem with item 2 of the Flow scale. This item in the clutch scale refers to the dimension called Automaticity of skills, during the subjective experience. This sense of automaticity is similar to that one that we are looking for in dimension 2 of flow, where the item states, “*things were happening automatically*”, whereas in the clutch item, this sense is described by “*I wasn’t thinking in the execution*”. Previous researchers have related those problems with the different experiences of the 9 dimensions to different causes across settings and depending on the type and kind of sports or individual differences (Swann et al., 2012). We understand that the specific cultural context related to groups can influence the interpretation of this subjective experience.

**TABLE 5:** Comparison among CFA measurement models (Clutch state scale).

Model	χ*^2^* (*df*)	*P*	RMSEA (CI 90%)	CFI	TLI
**Study 2**					
Original 6-item *Clutch State Scale*	30.649 (9)	<0.001	0.092 (0.057 –0.129)	0.954	0.924
Deletion of item 6 (Version 2)^1^	9.859 (4)	0.043	0.072 (0.012 –0.030)	0.987	0.967
**Study 3**					
Version 2	14.515 (4)	0.006	0.070 (0.034 –0.111)	0.985	0.961

^1^This model includes a correlated uniqueness between Item 1 and Item 2.

The measurement model without Item 6 showed a good fit to the data: χ^2^(*df*) = 9.859 (4), *p* = 0.043, RMSEA [90% CI] = 0.072 [0.012 –0.130], CFI = 0.987, TLI = 0.967. Such model also benefited from the inclusion of a correlated uniqueness between Items 1 and 2. Furthermore, all the items included in this model showed satisfactory factor loadings > 0.40 (range = 0.575–0.742; see Supplementary Table 2 in Supplementary material), no modification indices suggested further item deletion, and McDonald’s (1970) coefficient omega was excellent^1^ (Ω = 0.813). Once we finished this process, we labeled this 5-item, single-factor CSS as Version 2.

### Discussion

In Study 2, we began the process of validating the short forms developed in Study 1. Statistical evidence concerning their reliability, concurrent validity and improvement of data quality was obtained. Evidence supporting the single-factor structure of FSSS, and CSS was obtained from both exploratory and confirmatory approaches. Our results showed that two items presented fit problems, which coincides with previous research. The use of the different type of sports and cultural contexts have shown that these variations inside the sample can bring variation to the data results. This reinforces the need to continue studying both subjective states, looking for which dimensions can be described as universal, and which are more related to the individual characteristics inside the samples. A future study was then necessary for the last version of the short forms.

## Study 3: Psychometric validation and criterion validity

In Study 3, the last part of MIMO Phase IV, Evaluation of the Short Version, is applied. Study 3 has two main purposes: test the psychometric properties of FSSS-Version 2 and CSS-Version 2 and observe the relationship of flow and clutch with burnout and the subjective perception of performance.

### Participants

Sample 3 comprised 551 athletes, 382 men (72.07%) and 148 women (27.92%), with an age range of 16 to 35 years (*M* = 18.61, *SD* = 3.83) for both males and females (Men: *M* = 18.53, *SD* = 3.87; Women: *M* = 18.82, *SD* = 4.09). In this case, the sample is formed by different team sports. Football is repeated, and we introduce basketball, handball, volleyball, roller hockey, rugby and indoor football, and paddle and tennis as individual sports. The practice experience in training of the sample comprised from 1 to 24 years (*M* = 8.37, *SD* = 4.31; Men: *M* = 9.09, *SD* = 4.31; Women: *M* = 6.58, *SD* = 3.78). In addition, research on gender differences is maintained, since differences between men and women have been observed in other studies on optimal psychological states (Elbe et al., 2010; Chang, 2016).

#### Instruments

##### Short version of the Athlete Burnout Questionnaire (ABQ)

A short version of the original 15-item version of the ABQ developed by Arce et al. (2010) was used to measure burnout. De Francisco (2015) studied a 9-item version, in comparison with versions of 12 and 15 items of the same scale, finding that the model with nine was the one that presented a higher value of variance, in addition to a better fit in the confirmatory factor analysis and maintaining the levels of relationship between factors found in previous research (both in the original and in the Spanish version). This 9-item version was used as scale. The response format was Likert type, with four alternatives: “*Almost never*” (1), “*Rarely*” (2), “*Sometimes*” (3), “*Often*” (4).

##### Subjective perception of performance

The performance of the players was measured from two items adapted from the study of Bakker et al. (2011), who also wanted to observe the individual performance of football players. The response scale was adapted to a family scale within the culture of the study participants. From 1 to 10, in the first item, the participants had to assess how their performance was being “*given their abilities*”. And in the second, using the same 10-point scale, they had to judge their “*performances in competitive matches.*”

### Procedure

In this phase, the response to the questionnaires was done digitally, through Google Forms. The data was stored in a computer file and subsequently analyzed with the SPSS application. Confidentiality was guaranteed throughout.

### Criterion validity

Based on previous theoretical and empirical results, it is hypothesized that higher levels of experience of the optimal psychological states will reduce the possibility of developing burnout process in athletes, whereas greater experiences of the two optimal psychological states will produce an increase in the subjective perception of performance during sports practice. Finally, subjective perceptions of high performance will be negatively related to advanced states of burnout.

### Data analysis

Preliminary analyses included an examination of missing values, distribution of the data, and scale reliability using McDonald’s (1970) coefficient omega. Then, to obtain further evidence regarding the internal structure of the questionnaire, we used Mplus software version 7.4 with ML estimator (Muthén and Muthén, 2015) to confirm the single-factor structures described in Study 2.

To examine whether the FSSS-Version 2 and CSS-Version 2 displayed invariance across different groups of athletes (i.e., sex and age), we tested a series of models using Mplus 7.4. Specifically, we tested three models for each subdivision: configural (i.e., expected same factorial structure across groups), metric (i.e., expected same factor loadings across groups), and scalar (i.e., expected same item intercepts across groups) invariance. We compared those models by examining Satorra–Bentler χ^2^, CFI, TLI, and RMSEA differences. The more parsimonious model was selected only when changes in CFI were < 0.01 and increases in RMSEA were < 0.015 (Cheung and Rensvold, 2002; Chen, 2007). Changes in the TLI were evaluated following similar guidelines associated with changes in CFI (Marsh et al., 2009).

Finally, to obtain validity evidence based on relation to other variables of the FSSS-Version 2 and CSS-Version 2 scores, we tested two separate structural models. In those models, Flow and Clutch were expected to mediate the negative relationships between Burnout and Perceived performance. As a previous step before running the models, we first tested the measurement models of Burnout and Perceived stress. Burnout was expected to fit a 3-factor structure under an exploratory structural equation modeling (ESEM; Asparouhov and Muthén, 2009) approach and perceived stress to follow a single-factor structure. Then, we estimated the correlations among the latent factors of Burnout, Perceived performance, and Clutch/Flow. Finally, we included these factors in the two structural models to test the hypothesized relationships. Both structural models were tested using the ML estimator in Mplus software 7.4, and their fit was assessed using the same cut-off criteria indicated for the measurement models.

## Results

### Preliminary analysis and measurement models

Tables 6, 7 present the distribution of the data for the FSSS-Version 2 and CSS-Version 2 items. As can be observed, percentages of missing values were £ 0.4% for all the items, not causing any psychometric consequences on the data analyses (Graham, 2009). Omega reliability coefficients for both scales were excellent^1^ : Ω_Flow_ = 0.797, Ω_Clutch_ = 0.790.

**TABLE 6:** Data distribution and factor loadings of FSSS – Version 2 (Study 3).

	Data distribution (%)											l
Item	1	2	3	4	5	6	7	8	9	10	MV	
**Item 1**. *I felt I was competent enough to meet the high demands of the situation*	0.8	0.4	1.3	1.7	4.5	8.3	17.1	**32.5**	17.6	15.9	0.0	0.618
**Item 3.** *I had a strong sense of what I must do and what I wanted to achieve*	0.0	0.6	0.0	0.6	2.6	5.1	15.4	18.6	21.2	**36.0**	0.0	0.637
**Item 4**. *It was really clear to me that I was doing well*	0.2	0.8	1.7	3.4	6.2	9.4	22.9	**25.9**	12.4	17.1	0.2	0.710
**Item 5**. *I was completely focused on the task at hand*	0.2	0.2	0.6	1.5	2.8	6.6	16.9	24.2	20.3	**26.8**	0.0	0.662
**Item 6**. *I felt in total control of my mind and body*	0.2	0.2	2.6	3.0	4.3	8.8	15.9	**32.3**	17.1	15.6	0.0	0.636
**Item 7**. *I was not worried about what others may have been thinking of me*	3.0	3.4	5.1	5.6	7.3	11.8	10.3	13.1	13.9	**26.5**	0.0	0.564
**Item 9.** *I loved the feeling of that performance and want to capture it again*	0.2	0.2	0.4	0.8	2.6	5.6	10.1	15.8	17.6	**46.7**	0.0	0.428

The most selected category for each item is highlighted in bold. All factor loadings were significant at *p* < 0.001.

**TABLE 7:** Data distribution and factor loadings of CSS – Version 2 (Study 3).

	Data distribution (%)											l
Item	1	2	3	4	5	6	7	8	9	10	MV	
**Item 1**. *I was fully concentrated; I couldn’t have concentrated more; I was completely committed to what I need to do*	0.0	0.2	0.6	2.1	4.7	6.0	13.5	24.6	22.1	**26.3**	0.0	0.645
**Item 2.** *I was playing better because I was pushing to my limit consciously, giving my maximum*	0.2	0.4	0.8	2.8	1.7	9.4	13.7	24.8	20.3	**26.1**	0.0	0.700
**Item 3.** *Being more aware of my context and its relevance, I was thinking very clearly about me and what I was doing*	0.0	0.4	0.2	1.3	5.4	7.3	17.3	**28.5**	22.9	16.7	0.0	0.602
**Item 4.** *There was a full activation, with a mix of nerves and excitement, that pumped up my energy*	0.0	0.4	0.4	0.9	2.3	7.1	12.4	22.5	22.7	**31.0**	0.4	0.618
**Item 5.** *There’s no worrying about anything else, I was just focusing on the next move*	0.6	0.9	1.1	2.6	6.4	8.3	16.1	20.5	17.8	**25.5**	0.2	0.664

The most selected category for each item is highlighted in bold. All factor loadings were significant at *p* < 0.001.

Subsequently, we tested the measurement models of FSSS – Version 2 and CSS – Version 2 to confirm the structures refined in Study 2. On the one hand, the 7-item single-factor structure of FSSS – Version 2 presented an acceptable fit to the data: χ^2^(*df*) = 79.207 (14), *p* < 0.001, RMSEA [90% CI] = 0.093 [0.074–0.114], CFI = 0.932, TLI = 0.898. Moreover, all seven items presented satisfactory factor loadings > 0.40 (range = 0.428–0.710; see Table 4). On the other hand, the 5-item single-factor model of the CSS – Version 2 showed a good fit to the data: χ^2^(*df*) = 14.515 (4), *p* = 0.006, RMSEA [90% CI] = 0.070 [0.034–0.111], CFI = 0.985, TLI = 0.961. Again, all items presented satisfactory factor loadings (range = 0.602–0.700; see Table 5).

### Measurement invariance models of FSSS – Version 2 and CSS – Version 2

Tables 8, 9 present the goodness-of-fit indices for the measurement invariance models of FSSS – Version 2 and CSS – Version 2. On the one hand, for the CSS – Version 2, factorial structure, factor loadings, and item intercepts remained invariant across age and sex. On the other hand, however, invariance could not be assured for the FSSS – Version 2, as different indices pointed to different conclusions.

**TABLE 8:** Fit Indexes for the measurement invariance models of FSSS – Version 2 (Study 3).

Model	χ2(*df*)	RMSEA (90% CI)	CFI	TLI	Δχ2(Δ*df*)	*p*	Δ RMSEA	ΔCFI	ΔTLI
**Sex**									
Configural	132.097* (40)	0.093 (0.076–0.111)	0.903	0.865					
Metric	139.162* (47)	0.086 (0.070–0.103)	0.903	0.885	7.065 (7)	0.422	-0.007	0.000	0.020
Scalar	163.386* (54)	0.087 (0.072–0.103)	0.885	0.881	31.288 (14)	0.005	-0.005	-0.018	0.016
**Age**									
Configural	136.039* (40)	0.095 (0.078–0.113)	0.906	0.868					
Metric	154.522* (47)	0.093 (0.077–0.109)	0.894	0.874	18.483 (7)	0.010	0.002	-0.012	0.006
Scalar	165.646* (54)	0.088 (0.073–0.104)	0.890	0.886	29.607 (14)	0.009	-0.007	-0.016	0.018

The subsamples for the different measurement invariance analyses are Gender: n_female_ = 148, n_male_ = 382; Age: n_U18_ = 290, n_>18_ = 240;. *df* = degrees of freedom; RMSEA = root mean square error of approximation; CI = confidence interval; CFI = Comparative Fit Index; TLI = Tucker-Lewis Index.

*p < 0.001.

**TABLE 9:** Fit indexes for the measurement invariance models of CSS – Version 2 (Study 3).

Model	χ2(*df*)	RMSEA (90% CI)	CFI	TLI	Δχ2(Δ*df*)	*p*	Δ RMSEA	ΔCFI	ΔTLI
**Sex**									
Configural	45.372* (8)	0.133 (0.097–0.172)	0.948	0.869					
Metric	54.343* (12)	0.115 (0.085–0.147)	0.941	0.901	8.970 (4)	0.062	-0.003	0.000	0.004
Scalar	74.094* (16)	0.117 (0.091–0.145)	0.919	0.898	28.722 (8)	0.001	0.000	-0.007	-0.001
**Age**									
Configural	17.720* (8)	0.068 (0.024–0.111)	0.986	0.965					
Metric	23.814* (12)	0.061 (0.023–0.097)	0.983	0.972	6.093 (4)	0.192	-0.003	0.000	0.005
Scalar	28.386* (16)	0.054 (0.017–0.086)	0.982	0.978	10.666 (8)	0.221	-0.005	0.000	0.008

The subsamples for the different measurement invariance analyses are Gender: n_female_ = 148, n_male_ = 382; Age: n_U18_ = 290, n_>18_ = 240;. *df* = degrees of freedom; RMSEA = root mean square error of approximation; CI = confidence interval; CFI = Comparative Fit Index; TLI = Tucker-Lewis Index.

*p < 0.001.

### Validity evidence based on relation to other variables

#### Measurement model for burnout

Regarding the Burnout measurement model, the 3-factor ESEM showed a good fit to the data: χ^2^(*df*) = 22.066 (12), *p* = 0.037, RMSEA [90% CI] = 0.040 [0.010–0.065], CFI = 0.993, TLI = 0.978.

#### Structural models involving flow and clutch states

**FIGURE 1:**
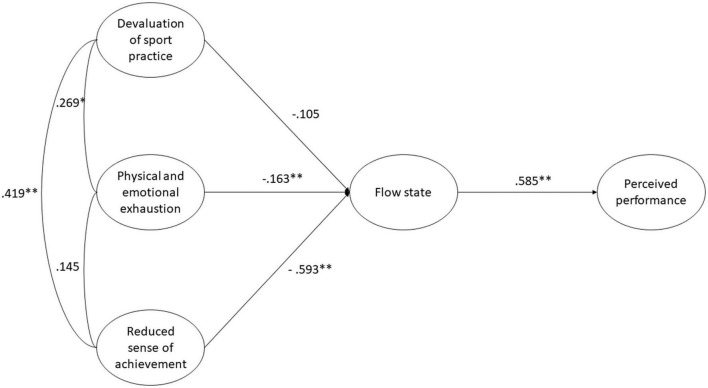
Structural equation model where players’ flow state mediates the relationship between burnout and perceived performance. *p < 0.05; **p < 0.001.

**FIGURE 2:**
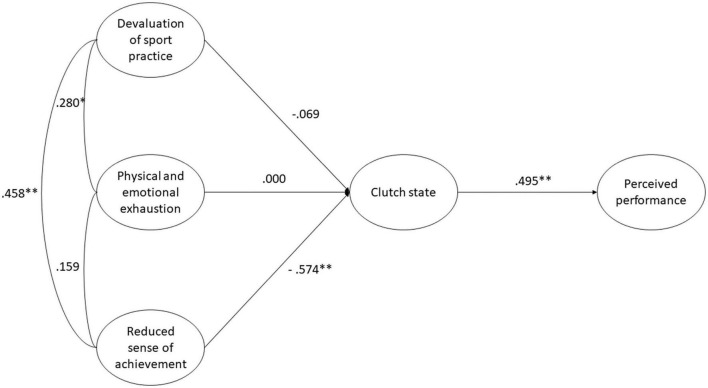
Structural equation model where players’ clutch state mediates the relationship between burnout and perceived performance. *p < 0.05; **p < 0.001.

As the final step of the analysis, we tested two separate structural equation models to assess the relationships of Flow and Clutch states with Burnout and Perceived performance. On the one hand, the model involving Flow showed a satisfactory fit to the data: χ^2^(*df*) = 283.404 (116), *p* < 0.001, RMSEA [90% CI] = 0.052 [0.044–0.060], CFI = 0.945, TLI = 0.928. As can be observed in Figure 1, physical and emotional exhaustion (β = -0.163) and Reduced sense of achievement (β = -0.593) negatively predicted players’ experience of the Flow state, which in turn was positively related to Perceived performance (β = 0.585). On the other hand, the model including Clutch also presented a satisfactory fit to the data: χ^2^(*df*) = 246.034 (84), *p* < 0.001, RMSEA [90% CI] = 0.060 [0.052–0.069], CFI = 0.940, TLI = 0.915. In this model (see Figure 2), players’ Reduced sense of achievement (β = -0.574) negatively predicted their experience of the Clutch state, which positively led to Perceived performance (β = 0.495).

### Discussion

In Study 3 we used the short version as a questionnaire, observing that the use of short versions reduces the missing data (£ 0.4%), improving the quality of the results. With good results in invariance testing for clutch, some statistical problems arise from flow, in line with previous research: sex (Elbe et al., 2010; Chang, 2016) and age (Jackson et al., 2008). As predicted, the experience of optimal psychological states increases the possibility of perceiving the own performance as better. At the same time, the repetition of the optimal psychological experiences, decreases the chances of being involved in burnout processes.

## General discussion

The research field of the optimal psychological states is “reaching a crisis point” (p.25, Swann et al., 2018) due to the accumulated anomalies and new insights that scientists are observing. Several critics can be seen in different systematic reviews about the flow topic (Swann et al., 2012; Goddard et al., 2021) where the classical nine-dimensions conceptualization have been put in doubt. For example, some authors find that only five dimensions of flow were experienced at a specific event (Swann et al., 2012), and others directly say that flow research has not produced a theory, and, for that, lacks explanatory power (Goddard et al., 2021). In this ongoing process of studying flow, the use of different methodologies (Swann et al., 2012, 2016a) produced the identification of what can be understood as a new and different optimal psychological state: clutch. As a new construct, today there are “significant definitional, conceptual, and measurement issues” (p. 24; Schweickle et al., 2020) that need to be studied. This was one of the purposes of this work, using the conceptualization for clutch of Swann et al. (2017a), and the principles of the Integrated Model of Flow and Clutch States (Swann et al., 2017b,^2019^), which describe the differences and similarities between both states. For this research, we studied both states at the same time, following the recommendations of Schweickle et al. (2020) applying the theory of The Integrated Model of Flow and Clutch States (Swann et al., 2017b,^2019^). FSSS and CSS were designed as single-factor instruments to capture the core aspects of each psychological state. In this way, evidence from both exploratory and confirmatory factor approaches supported the single-factor structure of the two short-forms.

We are attending to the growing demands for obtaining more functional and practical measurements in the sporting context (Ramis et al., 2010), in line with the idea that reducing application times in investigation and practice produce benefits to the research process (Jackson et al., 2008; Leo et al., 2015; García-Alba et al., 2021). With this objective, our new tools help in developing a better conceptual understanding and have several advantages related to their short forms: they can be efficient, place less burden on athletes and researchers, are economical, and help to improve the data quality (Alcaraz et al., 2020; Rey et al., 2021). For that purpose, we followed the MIMO strategy (Alcaraz et al., 2020) that comprises four phases: I) Initial selection of the items, II) Expert panel, III) Group focus and IV) Short form evaluation. Our work is respecting the three main goals of the MIMO process that says the new scales are in consonance with the actual literature, people that use them will be satisfied with the results, and they will be comprehensible for the participants that answer them.

In relation to flow, we found that the multifactorial model fit better with seven items than with nine, as it is in the classical Csikszentmihalyi definition. From the FSSS, Item 2, related to the union of action and awareness, and Item 8, related to the perception of time transformation, did not fit with the other dimensions. Differences in the perception of the subjective experience of flow have been shown for different authors and has been related to different causes as the diversity in the sports analyzed (Swann et al., 2012), gender (Elbe et al., 2010; Chang, 2016), or resources (Stander et al., 2015). Specifically for Item 2, in previous research in the Spanish context, poor results related to the union of attention and awareness have been seen. For example, López-Torres et al. (2007) found in swimmers that in their best performances only 20% of the athlete’s sample spoke about the experience of that dimension, and, on their bad days, no athlete mentioned that dimension. It seems that in the Spanish context, and more related to team sports, athletes do not identify their actions during the game as automatic. On the other hand, Item 8 of the flow scale appears to not completely identify the kind of perceptions athletes have related to the time experience. Problems in that dimension were indicated in previous research (Jackson and Marsh, 1996; García Calvo et al., 2008), and authors explained these differences based on sports modalities. Comparing swimmers and football players, it seems that for swimmers perceiving that they are experiencing flow, they would need to sense that the time passes more quickly. But football players, in the other hand, who must do fast movements to achieve success, in flow state they would be expecting that time passes more slowly (López-Torres et al., 2007; García Calvo et al., 2008). Accepting that sport modality will modulate the time expectancy that athletes have, and, coming out of the focus groups we organized, reflections of the athletes in team sport recognize two kinds of time experience while they are practicing sport. We can divide these differences into two levels: level 1 (*micro*), related to the moment where they are executing motor actions (usually a short period of time) and, level 2 (*macro*), a larger time related to the game competition or training session. With this double level of time perception, possibly one item cannot express the full experience of time perception. Thus, our 7-item scale seems to be more universally applicable, avoiding biases caused by differences in the type of sport and regional or cultural characteristics.

In the development of the CSS, we found some problems with Item 6, where its correlation with other factors was poor. This Item 6 of CSS together with Item 2 of FSSS are theoretically related in the term that both try to identify the sense of automaticity in the execution of the action. And, as seen before, some difficulties in previous research in Spanish context have been observed. And for the content of this item, it seems that there could be a relation with the same structural issue that shows time perception, based on a double level reality: a first level, where the individual focus is on the own action, and a second level, related to the actions of the team (individual actions in relation with the general coordination with the mates) during a specific event. Deleting that item, the results indicate that this new scale for studying clutch presents fit index values, optimal values of internal consistency, appropriate concurrent validity, and is invariant for gender and age.

Our results showed that these two states exist separately, and they are correlated. Moreover, the relation of flow and clutch with other psychological variables were in the expected direction, based on previous research (Bakker et al., 2011): positive for the subjective perception of players performance, and negative for burnout. Saying that, the flow short scale with seven items (FSSS – Version 2) has very good reliability, as is happening with the 5-item scale related to clutch (CSS – Version 2). On that point, these scales can be good tools to continue investigating the complex subjective experience of the optimal psychological states. The research in this field (reviewed in Schweickle et al., 2020; Goddard et al., 2021) brought us into a point where new scales were needed together with new insights into a better definition of flow and its relationship with what is already an optimal psychological state: clutch.

The study of the optimal psychological states brought researchers to the situation of describing two different states that represent similar experiences or that can be alternately experienced inside events but with specific characteristics. First, as validation of questionnaires is an ongoing process, future research should continue to assess and improve the short forms presented in this study to facilitate a better and deeper understanding of these optimal states. Specifically, further assessment of the invariance is required, as the FSSS – Version 2 failed to provide clear evidence supporting its invariance across age. In this line, maybe the experience of flow is conceptualized depending on the maturity level of the athlete. In addition, in this study, we focused on developing two efficient single-factor short forms that capture the core aspects of each optimal state. However, we understand that, as future research on those states evolves, new and more complex (i.e., multi-factorial) questionnaires would be required. Second, in our research, one limitation of the study is that the sample included mostly team sports. Another limitation is related to the lack of *a priori* sample power analysis. In our study, we relied on rules-of-thumb (e.g., Nunnally, 1967), but those rules could be outdated and thus we encourage future research to conduct an analysis of the sample size requirements before collecting the data (Wolf et al., 2013). And third, it would be interesting to use this short scale in different cultures to compare possible differences from regional contextualization about the two states.

This study shows the benefits of using short forms, relates to the percentage of missing data, and provides evidence supporting their psychometrical merit, obtained with a vast sporting sample. Together with that, we are continuing with the development of a better understanding of the subjective experiences related to optimal psychological states. In the breaking point that we are at in the field of flow research, new pathways are opened to continue researching the differences and similarities between the two optimal states: flow and clutch. These two instruments contribute a series of improvements in the understanding of the two optimal states, and, at the same time, these short forms versions also facilitate its uses in the professional sphere and its administration at different moments over the competition season. These two states are relevant for better sport practices as we have seen that they diminish the perception of burnout processes and increase the performance perception of the players.

## Data availability statement

The raw data supporting the conclusions of this article will be made available by the authors, without undue reservation.

## Ethics statement

The studies involving human participants were reviewed and approved by Comité de Ética de la Investigación. Written informed consent to participate in this study was provided by the participants’ legal guardian/next of kin.

## Author contributions

ASV, JC, and SA contributed to conception and design of the study. ASV collected the data and organized the database and wrote the first draft of the manuscript. JC and RM refined the design of the instruments. SA performed the statistical analysis. ASV and SA wrote sections of the manuscript. JC, SA, and RM contributed to manuscript revision, read, and approved the submitted version. All authors contributed to the article and approved the submitted version.

## Footnotes

1 We also provide evidence of the reliability of our scales by presenting the results of the alpha coefficients: α_Flow Study 2_ = 0.786, α_Flow Study 3_ = 0.791; α_Clutch Study 2_ = 0.812, α_Clutch Study 3_ = 0.786.
