# Supplementary material for: Measuring optimal psychological states: Proposal of two brief versions to measure flow and clutch in athletes
Source: Front Psychol. 2023 Jan 20;14:1066494. doi: 10.3389/fpsyg.2023.1066494 (PMC9895101; doi:10.3389/fpsyg.2023.1066494)
Supplement: Supplementary file 1 [file Data_Sheet_1.docx]

**Supplementary Materials**

**Table S1**

*Data Distribution and Factor Loadings of FSSS – Version 2 (Study 2).*

|  | Data distribution (%) | | | | | | | | | | | λ |
| --- | --- | --- | --- | --- | --- | --- | --- | --- | --- | --- | --- | --- |
| **Item** | 1 | 2 | 3 | 4 | 5 | 6 | 7 | 8 | 9 | 10 | MV |  |
| **Item 1**. *I felt I was competent enough to meet the high demands of the situation* | 0.0 | 0.0 | 0.3 | 2.4 | 4.2 | 11.2 | 22.0 | **25.9** | 15.0 | 18.5 | 0.3 | .629 |
| **Item 3.** *I had a strong sense of what I must do and what I wanted to achieve* | 0.0 | 0.0 | 0.7 | 0.7 | 3.5 | 5.6 | 9.4 | 26.2 | 21.7 | **31.5** | 0.7 | .670 |
| **Item 4**. *It was really clear to me that I was doing well* | 0.3 | 1.0 | 1.7 | 2.4 | 11.9 | 11.9 | 19.6 | **20.6** | 17.8 | 12.6 | 0.0 | .611 |
| **Item 5**. *I was completely focused on the task at hand* | 0.0 | 0.3 | 0.3 | 1.0 | 3.5 | 7.3 | 12.2 | 20.6 | **27.6** | 26.2 | 0.7 | .651 |
| **Item 6**. *I felt in total control of my mind and body* | 0.0 | 0.0 | 1.0 | 3.8 | 7.0 | 8.4 | 16.8 | **27.3** | 23.8 | 11.5 | 0.3 | .697 |
| **Item 7**. *I was not worried about what others may have been thinking of me* | 2.8 | 1.7 | 4.2 | 4.2 | 7.3 | 8.7 | 15.7 | 13.3 | 12.2 | **29.7** | 0.0 | .487 |
| **Item 9.** *I loved the feeling of that performance and want to capture it again* | 0.3 | 0.3 | 1.0 | 0.7 | 3.5 | 5.9 | 12.2 | 15.4 | 24.1 | **36.4** | 0.0 | .511 |

*Note.* The most selected category for each item is highlighted in bold. MV = Missing values.

All factor loadings were significant at *p* < .001.

**Table S2**

*Data Distribution and Factor Loadings of CSS – Version 2 (Study 2).*

|  | Data distribution (%) | | | | | | | | | | | λ |
| --- | --- | --- | --- | --- | --- | --- | --- | --- | --- | --- | --- | --- |
| **Item** | 1 | 2 | 3 | 4 | 5 | 6 | 7 | 8 | 9 | 10 | MV |  |
| **Item 1**. *I was fully concentrated; I couldn’t have concentrated more; I was completely committed to what I need to do* | 0.0 | 0.7 | 0.7 | 2.1 | 3.8 | 5.6 | 15.4 | 31.5 | 21.3 | 18.5 | 0.3 | .668 |
| **Item 2.** *I was playing better because I was pushing to my limit consciously, giving my maximum* | 0.0 | 0.7 | 0.7 | 1.4 | 4.2 | 3.8 | 18.2 | 23.1 | 26.2 | 21.0 | 0.7 | .742 |
| **Item 3.** *Being more aware of my context and its relevance, I was thinking very clearly about me and what I was doing* | 0.0 | 0.3 | 0.3 | 2.8 | 5.9 | 6.6 | 18.2 | 31.5 | 22.0 | 11.9 | 0.3 | .664 |
| **Item 4.** *There was a full activation, with a mix of nerves and excitement, that pumped up my energy* | 0.0 | 0.3 | 1.0 | 1.0 | 3.5 | 8.0 | 13.6 | 24.1 | 21.0 | 26.6 | 0.7 | .575 |
| **Item 5.** *There’s no worrying about anything else, I was just focusing on the next move* | 0.3 | 1.4 | 0.7 | 2.1 | 4.5 | 8.4 | 18.5 | 25.2 | 20.6 | 17.8 | 0.3 | .710 |

*Note.* The most selected category for each item is highlighted in bold. MV = Missing values.

All factor loadings were significant at *p* < .001.
